# 73204 HR the Silent Partner: Building Teams & Tools for Better Recruitment and Hiring of Clinical Research Professionals

**DOI:** 10.1017/cts.2021.512

**Published:** 2021-03-30

**Authors:** Elaine Fisher, Rebecca Thomas, Jeb Williams

**Affiliations:** 1 Emory University; 2 Emory Healthcare

## Abstract

ABSTRACT IMPACT: Improved non-biased matching of clinical research professionals to PI needs will accelerate time to active project engagement for new hires. OBJECTIVES/GOALS: An ongoing challenge for HR recruiters when matching applicants to open job positions is the time-consuming screening effort, which relies on imprecise semantic searching. We propose building a precision-based matching tool using Natural Language Processing to automate the accurate and non-biased identification of suitable job candidates. METHODS/STUDY POPULATION: We conducted 30-45’ interviews with HR administration/recruitment specialists to delineate the recruitment and hiring process used to match CRC resumes to job descriptions (n=7). Next, CRC applicant resumes were evaluated by experts, first by independent review, followed by consensus and assignment of a final rating, 0= not qualified; 1= CRC1; 2= CRC2; 3= CRC3; 4= CRC4. Guidelines evolved after reviewing 6 batches of 50 unique resumes (300 total) and were based on applicant qualifications & experiences by job level, CRC 1-4. Using final guidelines an additional 3,145 resumes were rated. For uniform input into the NLP model, resume formats were converted and text contents extracted into multiple sections, i.e., education, professional experiences, etc. RESULTS/ANTICIPATED RESULTS: Guideline development: Rater agreement improved over time with poor agreement when no guidelines were present (.161- Kappa) to good agreement for final guidelines (.608- Kappa). Spearman’s rho correlation between guideline iterations and Kappa is large and positive (rho 0.886) indicating significant rater agreement. NLP Model: Resume to job description matching indicated a third of applications were qualified, a third overqualified, and a third underqualified, suggesting the majority of applicants were unable to identify their ‘best fit’ by job level. Our NLP model matched the candidate resume to CRC level with 73.3% accuracy; and achieved 79.2% accuracy when matching the applicant resume to the CRC job description. Refinement of the NLP Model is ongoing. DISCUSSION/SIGNIFICANCE OF FINDINGS: A precision-based NLP matching tool will improve applicant targeting for the hire of great, qualified candidates. Improved applicant to job matching offers several advantages, i.e., reduced bias with greater diversity and inclusion; reduced time-to-hire; ability to anticipate training needs; and a reduced time to active project engagement.

